# Severe Septic Patients with Mitochondrial DNA Haplogroup JT Show Higher Survival Rates: A Prospective, Multicenter, Observational Study

**DOI:** 10.1371/journal.pone.0073320

**Published:** 2013-09-12

**Authors:** Leonardo Lorente, Ruth Iceta, María M. Martín, Esther López-Gallardo, Jordi Solé-Violán, José Blanquer, Lorenzo Labarta, César Díaz, Juan María Borreguero-León, Alejandro Jiménez, Julio Montoya, Eduardo Ruiz-Pesini

**Affiliations:** 1 Intensive Care Unit, Hospital Universitario de Canarias, La Laguna, Santa Cruz de Tenerife, Spain; 2 Departamento de Bioquímica y Biología Molecular y Celular, Centro de Investigaciones Biomédicas en Red de Enfermedades Raras (CIBERER), Universidad de Zaragoza, Zaragoza, Spain; 3 Intensive Care Unit, Hospital Universitario Nuestra Señora de Candelaria, Santa Cruz de Tenerife, Spain; 4 Intensive Care Unit, Hospital Universitario Dr, Negrín, Las Palmas de Gran Canaria, Spain; 5 Intensive Care Unit, Hospital Clínico Universitario, Valencia, Spain; 6 Intensive Care Unit, Hospital San Jorge, Huesca, Spain; 7 Intensive Care Unit, Hospital Insular, Las Palmas de Gran Canaria, Spain; 8 Laboratory Deparment, Hospital Universitario de Canarias, La Laguna, Santa Cruz de Tenerife, Spain; 9 Research Unit, Hospital Universitario de Canarias, La Laguna, Santa Cruz de Tenerife, Spain; 10 Fundación ARAID, Zaragoza, Spain; Ben-Gurion University of the Negev, Israel

## Abstract

**Objective:**

In a previous cohort study (n=96), we found an association between mitochondrial (mt) DNA haplogroup JT and increased survival of severe septic patients, after controlling for age and serum lactic acid levels. The aim of this research was to increase the predictive accuracy and to control for more confounder variables in a larger cohort (n=196) of severe septic patients, to confirm whether mtDNA haplogroup JT influences short and medium-term survival in these patients.

**Methods:**

We conducted a prospective, multicenter, observational study in six Spanish Intensive Care Units. We determined 30-day and 6-month survival and mtDNA haplogroup in this second cohort of 196 patients and in the global cohort (first and second cohorts combined) with 292 severe septic patients. Multiple logistic regression and Cox regression analyses were used to test for the association of mtDNA haplogroups JT with survival at 30-days and 6-months, controlling for age, sex, serum interleukin-6 levels and SOFA score.

**Results:**

Logistic and Cox regression analyses showed no differences in 30-day and 6-month survival between patients with mtDNA haplogroup JT and other haplogroups in the first cohort (n=96). In the second cohort (n=196), these analyses showed a trend to higher 30-day and 6-month survival in those with haplogroup JT. In the global cohort (n=292), logistic and Cox regression analyses showed higher 30-day and 6-month survival for haplogroup JT. There were no significant differences between J and T sub-haplogroups in 30-day and 6-month survival.

**Conclusions:**

The global cohort study (first and second cohorts combined), the largest to date reporting on mtDNA haplogroups in septic patients, confirmed that haplogroup JT patients showed increased 30-day and 6-month survival. This finding may be due to single nucleotide polymorphism defining the whole haplogroup JT and not separately for J or T sub-haplogroups.

## Introduction

Severe sepsis is a common, expensive, and frequently fatal condition [1,2]. Genetic factors may influence mortality in septic patients. Sepsis-related polymorphism studies have most commonly focused on genetic variants for specific genes whose protein products are elements of biologic pathways implicated in sepsis. These have included pro- and anti-inflammatory cytokines, innate immunity and coagulation/fibrinolysis pathways [3-5].

Although most genetic studies have focused on the immune system, recovery after sepsis is directly related to physiological reserve which is critically dependent on mitochondrial function [6]. Thirteen subunits of the mitochondrial oxidative phosphorylation system, the major pathway of cellular energy production, are coded in mitochondrial DNA (mtDNA). Interestingly, in 137 and 181 septic patients from a Chinese Han population, mtDNA macrolineage R was found to be a strong independent predictor of sepsis survival at 30 days [7] and six-months [8], respectively. In China, this macrolineage is found in approximately 35% of the population and mainly includes haplogroups B and F. In Europe, the macrolineage R includes haplogroups that are different from those of Asia. Thus, haplogroups HV, U and JT account for around 90% of the European population. Haplogroup HV contains haplogroup H. In 148 septic patients from England, haplogroup H was found to be a strong independent predictor of six-month outcome in patients with severe sepsis [6]. More recently, in 96 septic patients from Spain, we found that mtDNA haplogroup JT was associated with higher one-month survival, after controlling for age and serum lactic acid levels [9].

The aim of this research was to increase the predictive accuracy and to control for more confounder variables in a larger cohort (n=196) of severe septic patients, to confirm whether mtDNA haplogroup JT influences short and medium-term survival in these patients.

## Methods

### Design and subjects

A prospective, multicentre observational study was carried out in six Spanish intensive care units (ICUs). The Institutional Review Boards of the six hospitals approved this study: Hospital Universitario de Canarias (La Laguna. Santa Cruz de Tenerife, Spain), Hospital Universitario Nuestra Señora de Candelaria (Santa Cruz de Tenerife, Spain), Hospital Universitario Dr. Negrín (Las Palmas de Gran Canaria, Spain), Hospital Clínico Universitario de Valencia (Valencia. Spain), Hospital San Jorge (Huesca, Spain) and Hospital Insular (Las Palmas de Gran Canaria, Spain). Written informed consent from the patients or from their family members was obtained. We included patients with the diagnosis of severe sepsis according to the criteria of the International Sepsis Definitions Conference [10]. Exclusion criteria were: age < 18 years, pregnancy, lactation, infection by human immunodeficiency virus (HIV), white blood cell count < 1,000/mm^3^, solid or haematological tumour, or immunosuppressive, steroid or radiation therapy.

The following variables were recorded at baseline for each patient: gender, age, diabetes mellitus, chronic obstructive pulmonary disease (COPD), ischemic heart disease, ischemic stroke, site of infection, microorganism responsible, bloodstream infection, empiric antimicrobial therapy (this was considered adequate if the microorganism responsible of sepsis was susceptible at least to one antimicrobial agent used), mean blood pressure, septic shock, need and dose of norepinephrine, need and dose of dobutamine, pressure of arterial oxygen/fraction inspired (PaO_2_/FIO_2_), creatinine, bilirubin, leukocytes, lactic acid, platelets, international normalized ratio (INR), activated partial thromboplastin time (aPTT), interleukin-6, Acute Physiology and Chronic Health Evaluation (APACHE)-II score [11] and sepsis-related organ failure assessment (SOFA) score [12]. We used survival at 30 days and 6 months as endpoints.

### Haplogroup determination

**Figure 1:**
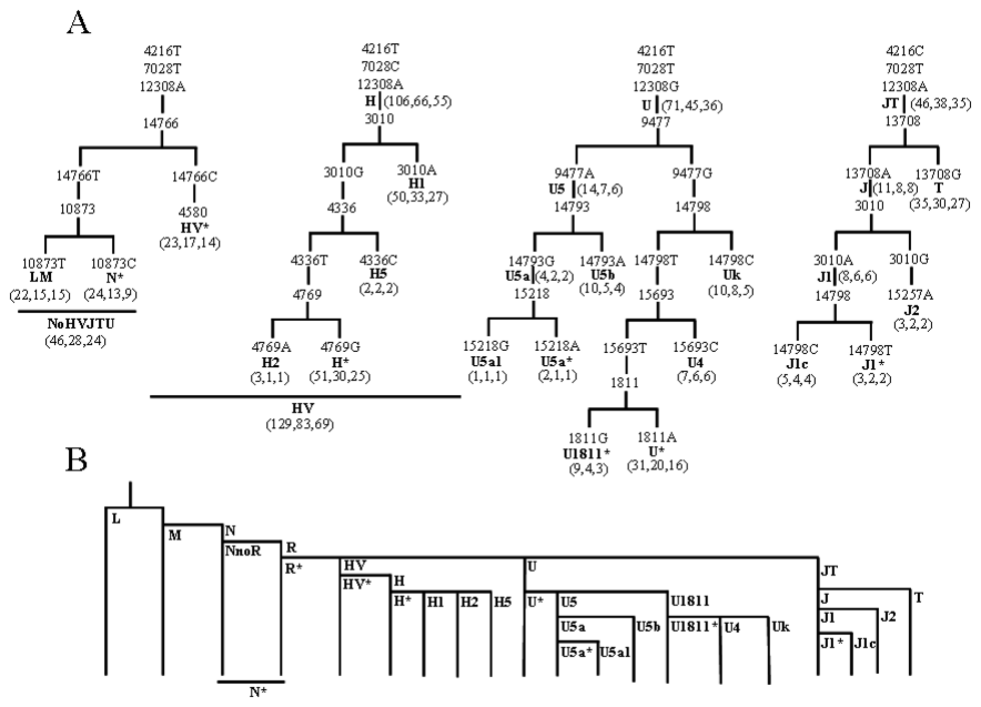
Mitochondrial DNA haplogroups. Haplogroup nomenclature is denoted in bold. N* defines those N non HV, JT or U individuals. HV* defines HV non H individuals. H* defines H non H1, H2 or H5 individuals. U5a* defines U5a non U5a1 individuals. U1811* defines U1811 non U4 or UK individuals. U* defines U non U4, UK, U1811* or U5 individuals. J1* defines J1 non J1c individuals. A) Haplogrouping strategy. The three numbers in brackets indicate the number of patients from each haplogroup and the number of one- and six-months survivors. B) Phylogeny summarizing the relationship between different mtDNA haplogroups.

Blood samples were collected from patients at the time of diagnosis of severe sepsis and frozen at -80°C. DNA was extracted following standard protocols and mtDNA haplogroup was determined by real time-polymerase chain reaction (RT-PCR). Three single nucleotide polymorphisms (SNPs) defining very frequent haplogroups JT (m.4216C), H (m.7028C) and U (m.12308G) were genotyped in all the samples. Fourteen other mtDNA SNPs (m.1811G, m.3010A, m.4336C, m.4580A, m.4769A, m.9477A, m.10873C, m.13708A, m.14766C, m.14793G, m.14798C, m.15218G, m.15257A, m.15693C) were analyzed using a hierarchic approach, taking into account haplogroup frequencies in the general Spanish population, to confirm particular haplogroups (Figure 1) [9]. The revised Cambridge Reference Sequence was used to define mtDNA position and nucleotide [13]. The RT-PCR was performed with TaqMan reagents (Applied Biosystems, Austin, TX). For each SNP, reagents included two primers around the SNP and two probes: a fluorophore vic-labelled probe specific for one allele and another fluorophore FAM-labelled probe specific for the other allele. DNA was amplified in a final volume of 25 µl, using 12.5 µl of TaqMan Gene Expression Master Mix, 0.9 µM of each primer, 0.2 µM of each probe and 10 ng of total DNA. The amplification was performed using universal conditions.

### Statistical analysis

We determined 30-days and 6-month survival and mtDNA haplogroup in the first cohort of 96 patients, second cohort of 196 patients and in the global cohort (first and second cohorts combined) with 292 severe septic patients.

Continuous variables are reported as medians and interquartile ranges, and were compared using Wilcoxon-Mann-Whitney test. Categorical variables are reported as frequencies and percentages, and were compared by chi-square test.

Multiple logistic regression and Cox regression analyses were used to test the association of mtDNA haplogroup JT with survival at 30 days and 6 months, controlling for age, sex, serum interleukin-6 levels and SOFA score. We used a step-by-step approach to select the variables included in the multivariable analysis; that is, we included in the final model those independent variables that allowed us to predict mortality. Odds Ratios and their 95% confidence intervals were calculated as measures of the association.

Survival analysis with Kaplan-Meier curve method and log-rank test comparisons were carried out using mtDNA haplogroup JT versus non-JT as the independent variable and survival at 30 days and 6 months as the dependent variable.

Differences with a P value of less than 0.05 were considered statistically significant. Statistical analyses were performed using SPSS 17.0 (SPSS Inc., Chicago, IL, USA) and Med Calc (Maria Kerke, Belgium).

## Results

In the global cohort (first and second cohort combined), patients with mtDNA haplogroup JT (n=46) showed significantly higher survival than patients with other haplogroups (n=246) at 30 days (82.6% vs 63.4%; p=0.007) and 6 months (76.1% vs 52.4%; p=0.002) (Table 1). Multiple logistic regression and Cox regression analyses showed that mtDNA haplogroup JT was associated with higher survival at 30-days or 6 months after controlling for age, sex, serum interleukin-6 levels and SOFA score (Table 2).

**Table 1:** Patients’ demographic and clinical characteristics according to mtDNA haplogroups of the 292 patients of global cohort (first and second cohort combined).

	HV (n=129)	U (n=71)	No R (n=46)	Total Non JT (n=246)	JT (n=46)	p JT vs Non JT
Gender male -n (%)	90 (69.8)	49 (69.0)	28 (60.9)	167 (67.9)	27 (58.7)	0.24
Age - median years (p 25-75)	61 (50-71)	61 (51-73)	61 (45-71)	61 (49-71)	57 (49-70)	0.42
Diabetes Mellitus -n (%)	42 (32.6)	22 (31.0)	10 (21.7)	74 (30.1)	10 (21.7)	0.29
COPD -n (%)	18 (14.0)	9 (12.7)	4 (8.7)	31 (12.6)	8 (17.4)	0.35
Ischemic heart disease -n (%)	15 (11.6)	7 (9.9)	5 (10.9)	27 (11.0)	3 (6.5)	0.44
Site of infection						0.51
- Respiratory -n (%)	78 (60.5)	43 (60.6)	23 (50.0)	144 (58.5)	23 (50.0)	
- Abdominal -n (%)	30 (23.3)	18 (25.3)	16 (34.8)	65 (26.4)	15 (32.6)	
- Neurological -n (%)	3 (2.3)	1 (1.4)	1 (2.2)	5 (2.0)	1 (2.2)	
- Urinary -n (%)	6 (4.6)	3 (4.2)	2 (4.3)	11 (4.5)	4 (8.7)	
- Skin -n (%)	4 (3.1)	2 (2.8)	4 (8.7) )	9 (3.7) )	3 (6.5)	
- Endocarditis -n (%)	7 (5.4)	4 (5.6)	0 (0)	11 (4.5)	0 (0)	
- Osteomyelitis -n (%)	1 (0.8)	0 (0)	0 (0)	1 (0.4)	0 (0)	
Microorganism responsibles						
- Unkwon -n (%)	66 (51.2)	37 (52.1)	23 (50.0)	126 (51.2)	20 (43.5)	0.42
- Gram-positive- n (%)	30 (23.3)	19 (26.8)	11 (23.9)	60 (24.4)	14 (30.4)	0.46
- Gram-negative- n (%)	31 (24.0)	16 (22.5)	12 (26.1)	59 (24.1)	12 (26.1)	0.85
- Fungii- n (%)	5 (3.9)	2 (2.8)	1 (2.2)	8 (3.3)	0 (0)	0.36
- Anaerobe- n (%)	1 (0.8)	0 (0)	1 (2.2)	2 (0.8)	1 (2.2)	0.40
Bloodstream infection	21 (16.3)	7 (9.9)	10 (21.7)	38 (15.4)	9 (19.6)	0.51
Empiric antimicrobial treatment adequate						0.58
- Unkown due to negative cultures- n (%)	66 (51.2)	36 (50.7)	23 (50.0)	125 (50.8)	20 (43.5)	
- Adequate -n (%)	49 (38.0)	30 (42.3)	22 (47.8)	101 (41.1)	20 (43.5)	
- Unkown due to antigenuria diagnosis- n (%)	3 (2.3)	2 (2.8)	1 (2.2)	6 (2.4)	2 (4.3)	
- Inadequate- n (%)	11 (8.5)	3 (4.2)	0 (0)	14 (5.7)	4 (8.7)	
Betalactamic more aminoglycoside- n (%)	28 (21.7)	15 (21.1)	10 (21.7)	53 (21.5)	11 (23.9)	0.70
Betalactamic more quinolone- n (%)	61 (47.3)	40 (56.3)	27 (58.7)	128 (52.0)	22 (47.8)	0.63
Septic shock- n (%)	110 (85.3)	60 (84.5)	40 (87.0)	210 (85.4)	41 (89.1)	0.65
PaO_2_/FIO_2_ ratio - median (p 25-75)	180 (121-257)	150 (86-250)	168 (93-328)	175 (106-260)	161 (104-252)	0.51
Creatinine (mg/dl) - median (p 25-75)	1.40 (0.85-2.50)	1.40 (0.90-2.00)	1.40 (0.70-2.60)	1.40 (0.90-2.35)	1.55 (0.97-2.85)	0.47
Bilirubin (mg/dl) - median (p 25-75)	1.00 (0.57-2.10)	0.90 (0.60-1.57)	0.85 (0.40-1.97)	0.94 (0.56-1.90)	1.30 (0.47-1.50)	0.56
Leukocytes -median*10^3^/mm^3^ (p 25-75)	15.2 (9.3-20.3)	11.9 (6.1-22.0)	12.8 (8.6-15.7)	14.0 (8.7-20.0)	15.9 (11.5-25.7)	0.89
Lactic acid - median mmol/L (p 25-75)	2.30 (1.20-4.55)	2.60 (1.40-4.20)	2.10 (0.90-4.70)	2.26 (1.20-4.40)	2.60 (1.25-4.05)	0.77
Platelets - median*103/mm^3^ (p 25-75)	176 (99-238)	192 (83-317)	196 (87-245)	179 (97-255)	145 (96-221)	0.17
INR - median (p 25-75)	1.40 (1.10-1.69)	1.29 (1.11-1.56)	1.33 (1.12-1.52)	1.33 (1.11-1.59)	1.24 (1.14-1.51)	0.56
aPTT - median seconds (p 25-75)	33 (28-43)	35 (29-41)	34 (28-50)	33 (28-43)	31 (27-42)	0.86
Interleukin-6 (pg/ml) - median (p 25-75)	110 (37-984)	171 (42-1070)	301 (45-1075)	161 (42-1012)	314 (32-1010)	0.59
SOFA score - median (p 25-75)	9 (7-12)	10 (8-12)	9 (7-12)	9 (7-12)	9 (7-12)	0.84
APACHE-II score - median (p 25-75)	20 (16-26)	20 (15-24)	21 (16-23)	20 (16-25)	20 (15-23)	0.52
Survivors at 30 days- n (%)	83 (64.3)	45 (63.4)	28 (60.9)	156 (63.4)	38 (82.6)	0.007
Survivors at 6 months- n (%)	69 (53.5)	36 (50.7)	24 (52.2)	129 (52.4)	35 (76.1)	0.002

COPD = chronic obstructive pulmonary disease; PaO_2_/FIO_2_ = pressure of arterial oxygen/fraction inspired oxygen; INR = International normalized ratio; aPTT = Activated partial thromboplastin time; SOFA = Sepsis-related Organ Failure Assessment; APACHE-II = Acute Physiology and Chronic Health Evaluation-II score. Data are presented as number (percentage) or median (interquartile range)

**Table 2:** Multiple logistic regression (MLR) and Cox regression analyses to predict mortality in the 292 patients of global cohort (first and second cohort combined).

	MLR OR (95% CI); p-value	Cox regression OR (95% CI); p-value
**Model: Mortality at 30 days in global cohort of 292 patients**		
mtDNA haplogroup JT	0.38 (0.15–0.95); 0.04	0.48 (0.22–0.97); 0.04
Serum Interleukin-6 levels	1.001 (1.0001-1.0015); 0.01	1.001 (1.0001-1.0015); <0.001
Age	1.16 (0.99–1.03); 0.16	1.16 (0.999–1.03); 0.05
Sex female	1.20 (0.66-2.19); 0.55	1.15 (0.73-1.79); 0.55
SOFA	1.15 (1.06–1.24); 0.001	1.15 (1.08–1.21); <0.001
**Model: Mortality at 6 months in global cohort of 292 patients**		
mtDNA haplogroup JT	0.40 (0.17–0.90); 0.03	0.51 (0.26–0.97); 0.04
Serum Interleukin-6 levels	1.0005 (0.999-1.001); 0.052	1.0005 (1.0001-1.001); 0.001
Age	1.02 (1.004–1.042); 0.02	1.02 (1.006–1.034); 0.005
Sex female	1.18 (0.67-2.09); 0.56	1.14 (0.77-1.70); 0.52
SOFA	1.15 (1.06–1.24); <0.001	1.12 (1.07–1.18); <0.001

OR = Odds Ratio; CI = Confidence Interval; SOFA = Sepsis-related Organ Failure Assessment

In the first cohort (n=96) we found no significant differences between patients with the mtDNA haplogroup JT (n=15) and other haplogroups (n=81) in survival at 30 days (86.7% vs 61.7%; p=0.17) and 6 months (73.3% vs 53.1%; p=0.08) (Table S1). Multiple logistic regression and Cox regression analyses did not show that mtDNA haplogroup JT was associated with higher survival at 30-days or 6 months, after controlling for age, sex, serum interleukin-6 levels and SOFA score (Table S3).

In the second cohort (n=196), patients with mtDNA haplogroup JT (n=31) showed a trend to higher survival at 30 days (80.6% vs 64.2%; p=0.054) and significantly higher survival at 6 months (77.4% vs 52.1%; p=0.007) than patients with other haplogroups (n=165) (Table S2). Multiple logistic regression and Cox regression analyses showed a non-significant association between mtDNA haplogroup JT and higher survival at 30 days or 6 months after controlling for age, sex, serum interleukin-6 levels and SOFA score (Table S3).

**Figure 2:**
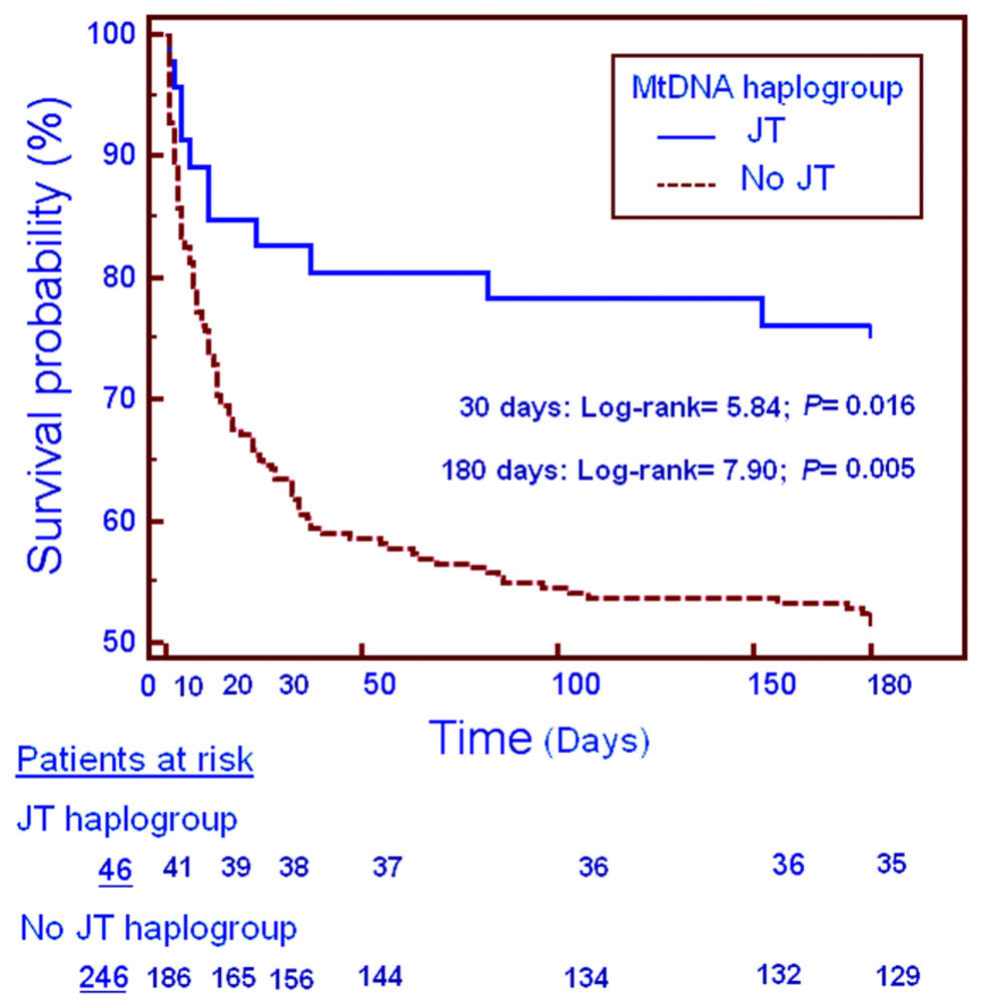
Cumulative proportion of patients' survival at 30 days and 6 months according to mtDNA haplogroup JT versus no JT.

Kaplan-Meier analysis confirmed a higher survival rate of patients with mtDNA haplogroup JT at 30 days (log-rank test=5.84; p=0.016) and 6 months (log-rank test=7.90; p=0.005) in the global cohort (Figure 2).

To check whether the association of haplogroup JT with a higher survival rate was due to SNPs defining this cluster or because of over-representation of one of the two sub-haplogroups that integrate the cluster, we separated our JT samples into sub-haplogroups J (n=11) and T (n=35). There were no significant differences between J and T sub-haplogroups in survival at 30 days [8/11 (72.7%) vs 30/35 (85.7%); p=0.37] and 6 months [8/11 (72.7%) vs 27/35 (77.1%); p=0.99]. Therefore, haplogroup JT-defining single nucleotide polymorphisms are probably responsible for the decreased risk of death from severe sepsis.

Since previously published work has shown that haplogroup H septic patients were more likely to survive to six months, we also studied survival of haplogroup H patients in our sample. There were no significant differences between haplogroup H and non-H patients in survival at 30 days [66/106 (62.3%) vs 128/186 (68.8%); p=0.30] and 6 months [55/106 (51.9%) vs 109/186 (58.6%); p=0.27]. In our sample these patients did not present a lower risk of early mortality.

Similarly, because previous publications have shown improved 6-month survival in haplogroup R septic patients, we also analyzed haplogroup R patient survival in our sample. Although 24 patients from N* lineages were not separated into R and non-R lineages, we found no significant differences between haplogroup R and non-R individuals in 30-day survival [166/246 (67.5%) vs 15/22 (68.2%); p=0.99] or 6-month survival [136/246 (55.3%) vs 15/22 (68.2%); p=0.27]. Thus in our sample, these patients did not present a lower risk of early mortality.

## Discussion

The global cohort study (first and second cohorts combined) is the largest to date reporting on mtDNA haplogroups in septic patients. The study confirmed that haplogroup JT septic patients showed increased 30-day and 6-month survival than non-JT haplogroup patients.

In addition, we previously found that sepsis patients surviving at least six-months had greater amounts of platelet cytochrome oxidase than those who died in this period [14,15] and that haplogroup JT patients had greater amounts of platelet cytochrome oxidase than patients with other mtDNA clusters [9]. As biologic plausibility is enhanced when a given polymorphism (haplogroup JT) is associated with a clinical phenotype (lower mortality due to severe sepsis) and an intermediate phenotype (higher cytochrome oxidase quantity) [4], all these results suggest that haplogroup JT modifies the risk of early death in septic patients. Because the survival rates were very similar between haplogroup JT sub-groups (sub-haplogroup J and sub-haplogroup T), then single nucleotide polymorphisms defining the whole haplogroup JT appear to be responsible for this association.

Four polymorphisms define haplogroup JT: m.4216C, m.11251G, m.15452A and m.16126C. The second is a synonymous polymorphism and does not change the amino acid in the polypeptide sequence and the last is located in the control region but it does not affect important regions involved in the regulation of replication or transcription. Therefore, m.4216TC, which provokes a p.MT-ND1:Y304H substitution, or m.15452CA, which produces a p.MT-CYB:L236I replacement, are the best candidates to explain these results. In m.15452CA there is a C to A transversion at mtDNA nucleotide position 15452 (m codes for mtDNA). In p.MT-CYB:L236I there is a replacement of leucine at cytochrome b amino acid position 236 by an isoleucine (p codes for protein). Interestingly, the m.4216T polymorphism, as in non-JT lineages, has been found to increase the risk of in-hospital mortality after severe injury in 525 white patients [16]. However, m.4216C, as in haplogroup JT, has also been shown to increase the risk of death in 136 patients after severe trauma [17]. In any case, and due to the complete linkage disequilibrium in the mtDNA, other SNPs linked to m.4216C could be responsible for the effect. In this scenario, the genotyping of only m.4216 position could produce opposing results. In fact, in addition to the European haplogroup JT, m.4216C has been also found defining Asian (D5c, M2a1b, M2c, M14), Australian (P4a), African (L4b1) and European (H10a) haplogroups [18]. Thus, the analysis of more than one haplogroup-defining SNP is necessary to target the causal polymorphism o combination of polymorphisms.

MtDNA genetic variation can alter the expression of nuclear genes [19]. Cybrids are cell lines that share the nuclear genetic background and culture conditions and only differ in the mtDNA genotype. Therefore, phenotypic differences between them must be due to the mtDNA genotype they harbour [20]. Very interestingly, it has been shown that, under stress conditions, IL-6 mRNA is more expressed in H than J cybrids [21]. Thus, these results suggest that, as haplogroup J expresses less IL-6 mRNA and IL-6 levels are positively associated with ICU mortality [22,23], the protective effect of mtDNA haplogroup JT against mortality by severe sepsis could be also through a lower production of this cytokine. In our study we found that serum IL-6 levels were associated with mortality. as previously described [22,23]. However, we found no differences in serum IL-6 levels between different mtDNA haplogroups.

The fact that we did not find any survival differences between R and non-R lineages of European septic patients is not surprising. MtDNA haplogroup R is defined by m.12705CT and m.16223CT polymorphisms. The first is a synonymous polymorphism and the second is located in the control region but it does not affect important regions involved in regulation or transcription. Therefore, the association between R lineages and sepsis survival in Chinese patients [7,8] must be due to an over-representation of R sub-haplogroups, such as B or F, that are not present in the European population.

A similar explanation cannot apparently be advanced for our different results in relation to haplogroup H. This association between haplogroup H and sepsis survival in English patients was for the whole haplogroup and not for particular sub-haplogroups [6]. It is unclear in this study how statistically significant confounders such as age, SOFA score and APACHE were controlled. However, in our study, patients with different haplogroups did not show differences in these possible confounders. In addition, we did not find significant differences in several parameters such as the causal microorganism of sepsis, the presence of bloodstream infection and the antimicrobials used, but these variables were not reported for the English patients [6].

Some limitations of this analysis should be recognized. For example, we only determined cytochrome oxidase quantity in 96 [9] out of the 292 patients examined in this work, although this is the only study on sepsis survival and mtDNA haplogroups that has included the measurement of an intermediate phenotype besides the association between genetic data and clinical phenotype. Sepsis survival probably depends on many different factors and, although our study represents the largest series reporting severe sepsis mortality according to mtDNA haplogroup, if we take into account these other factors then greater sample sizes should be considered for future studies.

## Conclusion

This global cohort study (first and second cohort combined) is the largest to date reporting on mtDNA haplogroups in septic patients. The study confirmed that haplogroup JT septic patients show increased 30-day and 6-month survival. This finding may be due to single nucleotide polymorphism defining the whole haplogroup JT and not separately for J or T sub-haplogroups.

## Supporting Information

Table S1 **Patients’demographic and clinical characteristics according to mtDNA haplogroups of the 96 patients of the first cohort.**
(DOC)

Table S2 **Patients’demographic and clinical characteristics according to mtDNA haplogroups of the 196 patients of the second cohort.**
(DOC)

Table S3 **Multiple logistic regression (MLR) and Cox regression analyses to predict mortality from first and second cohorts.**
(DOC)
