# Supplementary material for: Severe Septic Patients with Mitochondrial DNA Haplogroup JT Show Higher Survival Rates: A Prospective, Multicenter, Observational Study
Source: PLoS One. 2013 Sep 12;8(9):e73320. doi: 10.1371/journal.pone.0073320 (PMC3772099; doi:10.1371/journal.pone.0073320)
Supplement: Table S1 — Patients’demographic and clinical characteristics according to mtDNA haplogroups of the 96 patients of the first cohort. (DOC) [file pone.0073320.s001.doc]

**Table S1.** Patients’demographic and clinical characteristics according to mtDNA haplogroups of the 96 patients of the first cohort.

|  | HV  (n=44) | U  (n=24) | No R  (n=13) | Total  Non JT  (n=81) | JT  (n=15) | p  JT vs  Non JT |
| --- | --- | --- | --- | --- | --- | --- |
| Gender male – n (%) | 28 (63.6) | 17 (70.8) | 7 (53.8) | 52 (64.2) | 8 (53.3) | 0.56 |
| Age, years | 57 (46-65) | 62 (52-74) | 50 (39-66) | 59 (48-68) | 64 (54-81) | 0.11 |
| Diabetes Mellitus – n (%) | 21 (47.7) | 9 (37.5) | 2 (15.4) | 32 (39.5) | 4 (26.7) | 0.40 |
| COPD – n (%) | 5 (11.4) | 3 (12.5) | 1 (7.7) | 9(11.1) | 1 (6.7) | 0.99 |
| Ischemic heart disease – n (%) | 3 (6.8) | 2 (8.3) | 1 (7.7) | 6 (7.4) | 1 (6.7) | 0.99 |
| Site of infection |  |  |  |  |  | 0.96 |
| - Respiratory - n (%) | 31 (70.5) | 16 (66.7) | 7 (53.8) | 54 (66.7) | 8 (53.3) |  |
| - Abdominal - n (%) | 8 (18.2) | 4 (16.7) | 4 (30.8) | 16 (19.8) | 5 (33.3) |  |
| - Neurological - n (%) | 0 | 0 | 0 | 0 | 0 |  |
| - Urinary - n (%) | 1 (2.3) | 1 (4.2) | 1 (7.7) | 3 (3.7) | 1 (6.7) |  |
| - Skin - n (%) | 2 (4.5) | 2 (8.3) | 1 (7.7) | 5 (6.2) | 1 (6.7) |  |
| - Endocarditis - n (%) | 1 (2.3) | 1 (4.2) | 0 | 2 (2.5) | 0 |  |
| - Osteomyelitis - n (%) | 1 (2.3) | 0 | 0 | 1 (1.2) | 0 |  |
| Microorganism responsible |  |  |  |  |  |  |
| - Unkwon - n (%) | 23 (52.3) | 13 (54.2) | 7 (53.8) | 43 (53.1) | 6 (40.0) | 0.41 |
| - Gram-positive - n (%) | 11 (25.0) | 6 (25.0) | 2 (15.4) | 19 (23.5) | 5 (33.3) | 0.52 |
| - Gram-negative - n (%) | 9 (20.5) | 5 (20.8) | 4 (30.8) | 18 (22.2) | 4 (26.7) | 0.74 |
| - Fungii - n (%) | 3 (6.8) | 1 (4.2) | 0 | 4 (4.9) | 0 | 0.99 |
| - Anaerobe - n (%) | 1 (0.8) | 0 | 0 | 1 (1.2) | 0 | 0.99 |
| Bloodstream infection - n (%) | 5 (11.4) | 3 (12.5) | 2 (15.4) | 10 (12.3) | 3 (20.0) | 0.42 |
| Empiric antimicrobial treatment |  |  |  |  |  | 0.81 |
| - Unkown due to negative cultures- n (%) | 23 (52.3) | 12 (50.0) | 7 (53.8) | 42 (51.9) | 7 (46.7) |  |
| - Adequate - n (%) | 17 (38.6) | 10 (41.7) | 5 (38.5) | 32 (39.5) | 6 (40.0) |  |
| - Unkown due to antigenuria diagnosis- n (%) | 3 (6.8) | 2 (8.3) | 1 (7.7) | 6 (7.4) | 2 (13.3) |  |
| - Inadequate- n (%) | 1 (2.3) | 0 | 0 | 1 (1.2) | 0 |  |
| ß-lactamic more aminoglycoside-n (%) | 10 (22.7) | 5 (20.8) | 2 (15.4) | 17 (21.0) | 3 (20.0) | 0.30 |
| ß-lactamic more quinolone - n (%) | 25 (56.8) | 14 (58.3) | 7 (53.8) | 46 (56.8) | 6 (40.0) | 0.58 |
| Septic shock- n (%) | 38 (86.4) | 21 (87.5) | 11 (84.6) | 70 (86.4) | 13 (86.7) | 0.99 |
| PaO_2_/FIO_2_ ratio | 199 (107-280) | 152 (80-250) | 128 (99-335) | 160 (102-265) | 138 (84-197) | 0.98 |
| Creatinine, mg/dl | 1.20 (0.80-2.06) | 1.75 (1.00-2.17) | 0.75 (0.47-1.42) | 1.20 (0.70-2.00) | 2.05 (1.10-3.07) | 0.35 |
| Bilirubin, mg/dl | 1.20 (0.64-2.30) | 1.37 (0.88-2.56) | 0.40 (0.17-0.66) | 1.00 (0.55-2.30) | 0.92 (0.48-1.44) | 0.54 |
| Leukocytes *10^3^/mm^3^ | 12.2 (5.4-19.9) | 10.2 (5.2-20.4) | 13.8 (11.6-16.9) | 12.2 (5.4-19.9) | 16.4 (8.5-25.9) | 0.82 |
| Lactic acid, mmol/L | 2.35 (1.42-4.45) | 3.20 (1.30-5.87) | 1.30 (0.87-2.00) | 2.20 (1.30-4.37) | 3.80 (1.70-4.65) | 0.20 |
| Platelets *10^3^/mm^3^ | 166 (86-224) | 185 (78-312) | 207 (103-294) | 180 (89-266) | 120 (43-193) | 0.28 |
| INR | 1.42 (1.11-1.65) | 1.38 (1.20-1.65) | 1.21 (1.07-1.52) | 1.32 (1.11-1.56) | 1.24 (1.15-1.67) | 0.35 |
| aPTT, seconds | 33 (29-45) | 35 (31-41) | 30 (28-34) | 33 (29-42) | 36 (26-49) | 0.30 |
| Interleukin-6 (pg/ml) - median (p 25-75) | 199 (41-1043) | 387 (43-1149) | 84 (15-1317) | 155 (42-1051) | 345 (78-1083) | 0.51 |
| SOFA score | 9 (7-12) | 10 (9-14) | 9 (6-11) | 9 (7-12) | 9 (8-12) | 0.78 |
| APACHE-II | 20 (16-25) | 20 (14-24) | 23 (14-29) | 20 (16-25) | 21 (18-23) | 0.98 |
| Survivors at 30 days - n (%) | 26 (59.1) | 17 (70.8) | 7 (53.8) | 50 (61.7) | 13 (86.7) | 0.08 |
| Survivors at 6 months - n (%) | 23 (52.3) | 13 (54.2) | 7 (53.8) | 43 (53.1) | 11 (73.3) | 0.17 |

COPD = chronic obstructive pulmonary disease; PaO_2_/FIO_2_ = pressure of arterial oxygen/fraction inspired oxygen; INR = International normalized ratio; aPTT = Activated partial thromboplastin time; SOFA = Sepsis-related Organ Failure Assessment; APACHE-II = Acute Physiology and Chronic Health Evaluation-II score. Data are presented as number (percentage) or median (interquartile range)
