# Supplementary material for: Severe Septic Patients with Mitochondrial DNA Haplogroup JT Show Higher Survival Rates: A Prospective, Multicenter, Observational Study
Source: PLoS One. 2013 Sep 12;8(9):e73320. doi: 10.1371/journal.pone.0073320 (PMC3772099; doi:10.1371/journal.pone.0073320)
Supplement: Table S2 — Patients’demographic and clinical characteristics according to mtDNA haplogroups of the 196 patients of the second cohort. (DOC) [file pone.0073320.s002.doc]

**Table S2.** Patients’demographic and clinical characteristics according to mtDNA haplogroups of the 196 patients of the second cohort.

|  | HV  (n=85) | U  (n=47) | No R  (n=33) | Total  Non JT  (n=165) | JT  (n=31) | p  JT vs Non JT |
| --- | --- | --- | --- | --- | --- | --- |
| Gender male – n (%) | 62 (72.9) | 32 (68.1) | 21 (63.6) | 115 (69.7) | 19 (61.3) | 0.21 |
| Age - median years (p 25-75) | 65 (51-73) | 62 (48-69) | 65 (54-75) | 64 (50-72) | 54 (47-64) | 0.18 |
| Diabetes Mellitus – n (%) | 21 (24.7) | 13 (27.7) | 8 (24.2) | 42 (25.4) | 6 (19.3) | 0.36 |
| COPD – n (%) | 13 (15.3) | 6 (12.8) | 3 (9.1) | 22 (13.3) | 7 (22.6) | 0.27 |
| Ischemic heart disease - n (%) | 12 (14.1) | 5 (10.6) | 4 (12.1) | 21 (12.7) | 2 (6.4) | 0.71 |
| Site of infection |  |  |  |  |  | 0.63 |
| - Respiratory - n (%) | 47 (55.3) | 27 (57.4) | 16 (48.5) | 90 (54.6) | 15 (48.4) |  |
| - Abdominal - n (%) | 22 (25.9) | 14 (29.8) | 12 (36.4) | 49 (29.7) | 10 (32.3) |  |
| - Neurological - n (%) | 3 (3.5) | 1 (2.1) | 1 (3.0) | 5 (3.0) | 1 (3.2) |  |
| - Urinary - n (%) | 5 (5.9) | 2 (4.3) | 1 (3.0) | 8 (4.8) | 3 (9.7) |  |
| - Skin - n (%) | 2 (2.3) | 0 (0) | 3 (9.1)  ) | 4 (2.4)  ) | 2 (6.4) |  |
| - Endocarditis - n (%) | 6 (7.1) | 3 (6.4) | 0 (0) | 9 (5.4) | 0 (0) |  |
| - Osteomyelitis - n (%) | 0 (0) | 0 (0) | 0 (0) | 0 (0) | 0 (0) |  |
| Microorganism responsibles |  |  |  |  |  |  |
| - Unkwon - n (%) | 43 (50.6) | 24 (51.1) | 16 (48.5) | 83 (50.3) | 14 (52.2) | 0.24 |
| - Gram-positive- n (%) | 19 (22.3) | 13 (27.7) | 9 (27.3) | 41 (24.8) | 9 (29.0) | 0.65 |
| - Gram-negative- n (%) | 22 (25.9) | 11 (23.4) | 8 (24.2) | 41 (24.8) | 8 (25.8) | 0.26 |
| - Fungii- n (%) | 2 (2.3) | 1 (2.1) | 1 (3.0) | 4 (2.4) | 0 (0) | 0.68 |
| - Anaerobe- n (%) | 0 (0) | 0 (0) | 1 (3.0) | 1 (0.6) | 1 (3.2) | 0.29 |
| Bloodstream infection | 16 (18.8) | 4 (8.5) | 8 (24.2) | 28 (17.0) | 6 (19.3) | 0.19 |
| Empiric antimicrobial treatment adequate |  |  |  |  |  | 0.42 |
| - Unkown due to negative cultures- n (%) | 43 (50.6) | 24 (51.1) | 16 (48.5) | 83 (50.3) | 13 (41.9) |  |
| - Adequate - n (%) | 32 (37.7) | 20 (42.6) | 17 (51.5) | 69 (41.8) | 14 (45.2) |  |
| - Unkown due to antigenuria diagnosis- n (%) | 0 (0) | 0 (0) | 0 (0) | 0 (0) | 0 (0) |  |
| - Inadequate- n (%) | 10 (11.8) | 3 (6.4) | 0 (0) | 13 (7.9) | 4 (12.9) |  |
| Betalactamic more aminoglycoside- n (%) | 18 (21.2) | 10 (21.3) | 8 (24.2) | 36 (21.8) | 8 (25.8) | 0.34 |
| Betalactamic more quinolone- n (%) | 36 (42.3) | 26 (55.3) | 20 (60.6) | 82 (49.7) | 16 (51.6) | 0.56 |
| Septic shock- n (%) | 72 (84.7) | 39 (83.0) | 29 (87.9) | 140 (84.9) | 28 (90.3) | 0.42 |
| PaO_2_/FIO_2_ ratio - median (p 25-75) | 190 (135-270) | 175 (112-263) | 210 (102-353) | 180 (131-271) | 174 (120-260) | 0.43 |
| Creatinine (mg/dl) - median (p 25-75) | 1.50 (0.85-2.55) | 1.35 (0.90-2.30) | 1.60 (1.07-2.85) | 1.50 (0.90-2.50) | 1.45 (0.90-2.52) | 0.91 |
| Bilirubin (mg/dl) - median (p 25-75) | 1.04 (0.60-2.27) | 0.80 (0.60-1.27) | 1.35 (0.41-2.74) | 0.90 (0.60-2.00) | 1.50 (0.48-1.60) | 0.59 |
| Leukocytes -median*10^3^/mm^3^ (p 25-75) | 15.9 (11.0-21.7) | 15.4 (9.6-20.9) | 12.1 (8.7-17.6) | 15.1 (9.6-20.3) | 15.6 (6.9-23.3) | 0.71 |
| Lactic acid - median mmol/L (p 25-75) | 2.00 (1.20-3.80) | 2.35 (1.40-3.72) | 2.35 (1.32-5.90) | 2.30 (1.30-4.30) | 2.00 (1.30-4.60) | 0.91 |
| Platelets - median*10^3^/mm^3^ (p 25-75) | 188 (100-268) | 178 (107-280) | 175 (86-240) | 179 (99-260) | 156 (99-230) | 0.49 |
| INR - median (p 25-75) | 1.34 (1.10-1.80) | 1.31 (1.16-1.65) | 1.36 (1.19-1.59) | 1.34 (1.10-1.65) | 1.24 (1.11-1.41) | 0.18 |
| aPTT - median seconds (p 25-75) | 32 (28-43) | 35 (29-44) | 36 (27-54) | 34 (28-44) | 29 (27-34) | 0.61 |
| Interleukin-6 (pg/ml) - median (p 25-75) | 101 (29-726) | 170 (39-705) | 468 (68-1108) | 165 (39-851) | 309 (23-917) | 0.93 |
| SOFA score - median (p 25-75) | 10 (7-12) | 10 (7-13) | 9 (7-14) | 10 (7-12) | 10 (7-12) | 0.64 |
| APACHE-II score - median (p 25-75) | 20 (15-26) | 20 (15-25) | 21 (16-23) | 20 (15-25) | 20 (15-23) | 0.44 |
| Survivors at 30 days- n (%) | 57 (67.1) | 28 (59.6) | 21 (63.6) | 106 (64.2) | 25 (80.6) | 0.054 |
| Survivors at 6 months- n (%) | 46 (54.1) | 23 (48.9) | 17 (51.5) | 86 (52.1) | 24 (77.4) | 0.007 |

COPD = chronic obstructive pulmonary disease; PaO_2_/FIO_2_ = pressure of arterial oxygen/fraction inspired oxygen; INR = International normalized ratio; aPTT = Activated partial thromboplastin time; SOFA = Sepsis-related Organ Failure Assessment; APACHE-II = Acute Physiology and Chronic Health Evaluation-II score. Data are presented as number (percentage) or median (interquartile range)
