# Supplementary material for: Severe Septic Patients with Mitochondrial DNA Haplogroup JT Show Higher Survival Rates: A Prospective, Multicenter, Observational Study
Source: PLoS One. 2013 Sep 12;8(9):e73320. doi: 10.1371/journal.pone.0073320 (PMC3772099; doi:10.1371/journal.pone.0073320)
Supplement: Table S3 — Multiple logistic regression (MLR) and Cox regression analyses to predict mortality from first and second cohorts. (DOC) [file pone.0073320.s003.doc]

**Table S3.** Multiple logistic regression (MLR) and Cox regression analyses to predict mortality from first and second cohorts.

|  | MLR  OR (95% CI); p-value | Cox regression  OR (95% CI); p-value |
| --- | --- | --- |
|  |  |  |
| **Model: Mortality at 30 days**  **in first cohort of 96 patients** |  |  |
| mtDNA haplogroup JT | 0.20 (0.03–1.27); 0.09 | 0.34 (0.08–1.55); 0.17 |
| Serum Interleukin-6 levels | 1.001 (0.999-1.0015); 0.31 | 1.001 (0.999-1.0015); 0.34 |
| Age | 1.03 (0.997–1.07); 0.07 | 1.03 (0.999–1.06); 0.06 |
| Sex female | 2.01 (0.72-5.57); 0.18 | 1.80 (0.84-3.86); 0.13 |
| SOFA | 1.21 (1.03–1.42); 0.02 | 1.17 (1.04–1.30); 0.006 |
| **Model: Mortality at 6 months**  **in first cohort of 96 patients** |  |  |
| mtDNA haplogroup JT | 0.43 (0.10–1.88); 0.26 | 0.53 (0.18–1.60); 0.26 |
| Serum Interleukin-6 levels | 1.001 (0.999-1.0015); 0.39 | 1.001 (0.999-1.0015); 0.30 |
| Age | 1.04 (1.002–1.07); 0.03 | 1.03 (1.003–1.05); 0.03 |
| Sex female | 1.75 (0.68-4.51); 0.25 | 1.63 (0.83-3.19); 0.15 |
| SOFA | 1.14 (0.98–1.33); 0.09 | 1.13 (1.02–1.25); 0.02 |
| **Model: Mortality at 30 days**  **in second cohort of 196 patients** |  |  |
| mtDNA haplogroup JT | 0.45 (0.15–1.18); 0.07 | 0.51 (0.20–1.30); 0.16 |
| Serum Interleukin-6 levels | 1.001 (1.0001-1.0015); 0.02 | 1.001 (1.0001-1.0015); 0.001 |
| Age | 1.01 (0.98–1.03); 0.56 | 1.01 (0.993–1.03); 0.21 |
| Sex female | 0.91 (0.42-1.97); 0.81 | 0.94 (0.53-1.70); 0.85 |
| SOFA | 1.18 (1.07–1.29); 0.001 | 1.14 (1.06–1.22); <0.001 |
| **Model: Mortality at 6 months**  **in second cohort of 196 patients** |  |  |
| mtDNA haplogroup JT | 0.36 (0.13–1.002); 0.050 | 0.45 (0.19–1.04); 0.06 |
| Serum Interleukin-6 levels | 1.001 (0.999-1.0015); 0.09 | 1.001 (1.0001-1.0015); 0.003 |
| Age | 1.02 (0.99–1.04); 0.16 | 1.02 (0.999–1.03); 0.06 |
| Sex female | 0.95 (0.46-1.97); 0.90 | 0.96 (0.58-1.60); 0.89 |
| SOFA | 1.15 (1.05–1.26); 0.002 | 1.12 (1.05–1.19); <0.001 |

OR=Odds Ratio; CI=Confidence Interval; SOFA=Sepsis-related Organ Failure Assessment
